# New Insights Into Cranial Synchondrosis Development: A Mini Review

**DOI:** 10.3389/fcell.2020.00706

**Published:** 2020-08-11

**Authors:** Noriko Funato

**Affiliations:** ^1^Department of Signal Gene Regulation, Tokyo Medical and Dental University, Tokyo, Japan; ^2^Research Core, Tokyo Medical and Dental University, Tokyo, Japan

**Keywords:** cranial base, cartilage, mesoderm, neural crest, spheno-occipital synchondrosis, intersphenoid synchondrosis, *RUNX2*

## Abstract

The synchondroses formed via endochondral ossification in the cranial base are an important growth center for the neurocranium. Abnormalities in the synchondroses affect cranial base elongation and the development of adjacent regions, including the craniofacial bones. In the central region of the cranial base, there are two synchondroses present—the intersphenoid synchondrosis and the spheno-occipital synchondrosis. These synchondroses consist of mirror image bipolar growth plates. The cross-talk of several signaling pathways, including the parathyroid hormone-like hormone (PTHLH)/parathyroid hormone-related protein (PTHrP), Indian hedgehog (Ihh), Wnt/β-catenin, and fibroblast growth factor (FGF) pathways, as well as regulation by cilium assembly and the transcription factors encoded by the *RUNX2*, *SIX1*, *SIX2*, *SIX4*, and *TBX1* genes, play critical roles in synchondrosis development. Deletions or activation of these gene products in mice causes the abnormal ossification of cranial synchondrosis and skeletal elements. Gene disruption leads to both similar and markedly different abnormalities in the development of intersphenoid synchondrosis and spheno-occipital synchondrosis, as well as in the phenotypes of synchondroses and skeletal bones. This paper reviews the development of cranial synchondroses, along with its regulation by the signaling pathways and transcription factors, highlighting the differences between intersphenoid synchondrosis and spheno-occipital synchondrosis.

## Introduction

In vertebrates, the cranial base lies below the brain and forms a central bone structure of the skull. Within the cranial base, synchondroses play a critical role in the longitudinal growth of the skull (McBratney-Owen et al., 2008; Wei et al., 2017). Precocious ossification and/or malformation of cranial synchondroses can induce the fusion of adjacent bones and subsequent cranium deformities, such as microcephaly and midface hypoplasia (Goldstein et al., 2014; Funato et al., 2020). In order to understand the nature of craniofacial development and related congenital anomalies, identifying the signaling molecules that regulate synchondrosis development is necessary.

**FIGURE 1:**
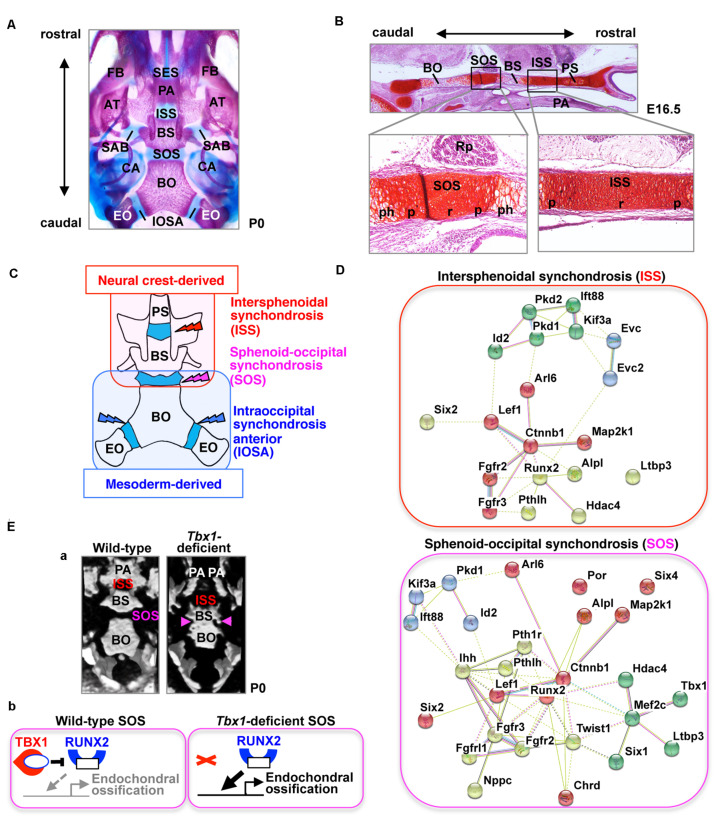
The cranial base and synchondroses. **(A)** Ventral view of bone staining of the mouse cranial base at postnatal day (P) 0. The middle line of the cranial base is formed by the presphenoid, basisphenoid (BS), and basioccipital (BO) bones. Between the mineralized bones, there are two cartilaginous synchondroses, the intersphenoid synchondrosis (ISS) and the spheno-occipital synchondrosis (SOS). Please note that the presphenoid bone is invisible because of the palatine process (PA). AT, ala temporalis (greater wing) of the basisphenoid bone; CA, canalicular part of auditory capsule; EO, exoccipital bone; FB, facial bone; IOSA, intraoccipital synchondrosis; SAB, synchondrosis, alar-basisphenoidalis; SES, spheno-ethmoidal synchondrosis. **(B)** Safranin-O staining of the mouse cranial base at embryonic day (E) 16.5. The presphenoid (PS), basisphenoid (BS), and basioccipital (BO) bones are separated by two synchondroses, the intersphenoid synchondrosis (ISS) and spheno-occipital synchondrosis (SOS). The synchondrosis is composed of bipolar growth plates with a central resting (r), proliferating (p), and prehypertrophic (ph) zones. PA, palate; Rp, Rathke’s pouch. **(C)** Schematic illustration of the tissue origins of the cranial base derived from the neural crest shown in red and those derived from the mesoderm in blue (McBratney-Owen et al., 2008). BS, basisphenoid bone; BO, basioccipital bone; EO, exoccipital bone; PS, presphenoid bone. **(D)** STRING protein-protein interaction network of mouse genes involved in abnormal synchondroses. The network was constructed using the STRING tool^3^, with mouse genes involved in abnormal synchondroses (Table 1) used as input. Different colors represent different kinds of evidence of connection between proteins. **(E) (a)** Skulls from wild-type and *Tbx1*-deficient mice at birth were analyzed by micro-computed tomography and are shown in a “bird’s eye view.” In *Tbx1*-deficient mice, the spheno-occipital synchondrosis (SOS) was completely mineralized (Funato et al., 2020). BO, basioccipital bone; BS, basisphenoid bone; ISS, intersphenoid synchondrosis; PA, palatine process. **(b)** A predicted model for TBX1-mediated regulation of endochondral ossification of SOS. By inhibiting the activity of RUNX2 and the expression of RUNX2 target genes, TBX1 negatively regulates chondrocyte differentiation as well as subsequent endochondral ossification in the SOS.

Cartilaginous segments that persist between the ossification centers in the cranial base represent various synchondroses, such as the sphenoethmoidal synchondrosis, intersphenoid synchondrosis (ISS), spheno-occipital synchondrosis (SOS), and intraoccipital synchondrosis (Figure 1A). The ISS is located between the presphenoid and basisphenoid bones in the central region of the cranial base, while the SOS is located between the basisphenoid and basioccipital bones (Figures 1A,B). The medial line of the cranial base is originally composed of the hypophyseal, acrochordal, and parachordal cartilages (McBratney-Owen et al., 2008). Subsequently, the hypophyseal cartilage and acrochordal cartilage develop into the ISS and SOS, respectively (McBratney-Owen et al., 2008). The cartilage primordium of the ISS is derived from the neural crest, whereas the SOS has a more complex origin, wherein its cartilage primordium is derived from the neural crest as well as the cranial mesoderm (Figure 1C; McBratney-Owen et al., 2008). The SOS contributes to the embryonic and postnatal elongation of the cranial base, until its ossification between the ages of 16 and 18 years in humans, whereas complete ossification of the ISS occurs between 2 and 3 years of age (Madeline and Elster, 1995), suggesting that the role of the SOS, in particular, is important in the postnatal stage.

The cranial base is formed by endochondral ossification, which begins with the formation of a cartilage primordium from condensed mesenchymal cells (McBratney-Owen et al., 2008). Chondrocyte proliferation maintains the synchondroses and leads to elongation of the cranial base (Matsushita et al., 2009). Immature chondrocytes undergo hypertrophy and subsequent apoptosis, followed by the formation of ossification centers after the invasion of osteoblasts from the perichondrium (St-Jacques et al., 1999). Endochondral ossification of the cranial synchondroses is different from that of skeletal bones in several ways. The synchondrosis is composed of bipolar growth plates with resting, proliferating, pre-hypertrophic, and hypertrophic zones that produce growth in opposing directions, whereas long bones are composed of a unipolar growth plate (Wei et al., 2016). This review presents new insights on the signaling pathways and transcription factors involved in the regulation of synchondrosis development, highlighting the differences and similarities between synchondroses present in the cranial base.

## Signaling Pathways in Synchondrosis Development

For the normal progression of the development of synchondroses, stringent regulation of chondrocyte differentiation in the cranial synchondroses is crucial. To find the relationship between genetic or molecular interaction networks in the synchondroses, genetically modified mice associated with abnormal synchondroses were investigated. Using the Mouse Genome Informatics^1^ database and PubMed^2^, 31 mouse genes with abnormal annotations in SOS and/or ISS were discovered (Table 1). These genes indicated that the regulation of synchondrosis development involves the interaction of several signaling pathways, including the parathyroid hormone-like hormone (PTHLH)/parathyroid hormone-related protein (PTHrP), Indian hedgehog (Ihh), Wnt/β-catenin, and fibroblast growth factor (FGF) pathways, as well as control by cilium assembly and by transcription factors encoded by specific genes (Figure 1D). This review focuses on the genes listed in Table 1.

**TABLE 1:** Mouse genes involved in abnormal development of the cranial synchondroses.

Gene	Protein	Induced mutation type	SOS/ISS	Age	Ossification	References
*Tbx1*	T-box 1	Deletion (*Mesp1-Cre*)	SOS	E15.5	Partially increased	Funato et al., 2020
*Fgfrl1*	FGF receptor like 1	Deletion	SOS	E18.5	Increased	Catela et al., 2009
*Ihh*	Indian hedgehog	Deletion (*Col2a1-Cre*)	SOS	E18.5	Increased	Razzaque et al., 2005
*Mef2c*	myocyte enhancer factor 2C	Activation	SOS	E18.5	Increased	Arnold et al., 2007
*Pth1r*	parathyroid hormone 1 receptor	Deletion	SOS	E18.5	Increased	Lanske et al., 1996
*Six1*; *Six4*	sine oculis-related homeobox 1; sine oculis-related homeobox 4	Deletion	SOS	E18.5	Partially increased	He et al., 2010
*Chrd*	chordin	Deletion	SOS	P1	Partially increased	Bachiller et al., 2003
*Por*	P450 oxidoreductase	Deletion (*Dermo1-Cre*)	SOS	P4	Partially increased	Panda et al., 2013
*Nppc*	natriuretic peptide type C	Deletion	SOS	P14	Decreased	Nakao et al., 2013
*Twist1*	twist bHLH transcription factor 1	Deletion (heterozygous)	SOS	P25-30	Increased	Hermann et al., 2012
*Evc*	EvC ciliary complex subunit 1	Deletion	ISS	E18.5	Increased	Pacheco et al., 2012
*Evc2*	EvC ciliary complex subunit 2	Deletion	ISS	E18.5	Increased	Caparrós-Martín et al., 2013
*Pkd2*	polycystin 2	Deletion (*Wnt1-Cre*)	ISS	P14	Increased	Khonsari et al., 2013
*Lef1*	lymphoid enhancer binding factor 1	Activation	ISS, SOS	E17.5	Increased	Nagayama et al., 2008
*Ctnnb1*	catenin beta 1	Deletion (*Col2a1-Cre*)	ISS, SOS	E17.5	Decreased	Nagayama et al., 2008
*Arl6/Bbs3*	ADP-ribosylation factor-like 6	Deletion	ISS, SOS	E18.5	Decreased	Kawasaki et al., 2017
*Pthlh/Pthrp*	parathyroid hormone-like peptide	Deletion	ISS, SOS	P1	Increased	Ishii-Suzuki et al., 1999
*Runx2*	runt-related transcription factor 2	Activation	ISS, SOS	P1	Increased	Takeda et al., 2001
*Six2*	sine oculis-related homeobox 2	Deletion	ISS, SOS	P1	Increased	He et al., 2010
*Fgfr2*	fibroblast growth factor receptor 2	Activation	ISS, SOS	P1 (SOS) P28 (ISS)	Increased	Nagata et al., 2011
*Pkd1*	polycystin 1	Deletion (*Dermo1-Cre*)	ISS, SOS	P5	Partially increased	Kolpakova-Hart et al., 2008
*Kif3a*	kinesin family member 3A	Deletion (*Col2a1-Cre*)	ISS, SOS	P7	Partially increased	Koyama et al., 2007
*Hdac4*	histone deacetylase 4	Deletion	ISS, SOS	P8	Increased	Vega et al., 2004
*Map2k1*	mitogen-activated protein kinase kinase 1	Activation	ISS, SOS	P11	Increased	Matsushita et al., 2009
*Id2*	inhibitor of DNA binding 2	Deletion	ISS, SOS	P14	Growth defects	Sakata-Goto et al., 2012
*Ift88*	intraflagellar transport 88	Deletion (*Col2a1-Cre*)	ISS, SOS	P14	Partially increased	Ochiai et al., 2009
*Alpl*	alkaline phosphatase, liver/bone/kidney	Deletion	ISS, SOS	P20	Increased	Liu et al., 2014; Nam et al., 2017
*Ltbp3*	latent transforming growth factor beta binding protein 3	Deletion	ISS, SOS	P21	Increased	Dabovic et al., 2002
*Fgfr3*	fibroblast growth factor receptor 3	Activation	ISS, SOS	P21	Increased	Chen et al., 1999
*Pfas*	Phosphoribosyl-formylglycinamidine synthase	Mutation (heterozygous)	n/a	P84	Increased	Palmer et al., 2016

*E, embryonic day; P, postnatal day; SOS, spheno-occipital synchondrosis; ISS, intersphenoid synchondrosis; n/a, not available.*

### Runt-Related Transcription Factor 2

Runt-Related Transcription Factor 2 (*RUNX2*), a gene implicated in cleidocranial dysplasia (Online Mendelian Inheritance in Man; OMIM #119600), is a crucial transcription factor of osteoblast and chondrocyte differentiation (Ducy et al., 1997; Komori et al., 1997; Yoshida et al., 2004). Skull radiography of patients with cleidocranial dysplasia caused by *RUNX2* haploinsufficiency showed persistent synchondroses primarily associated with defective development of membranous bones (Kreiborg et al., 1999; Al Kaissi et al., 2013). Chondrocyte-specific constitutive *Runx2* expression in mice has also been shown to induce precocious endochondral ossification in the cranial cartilage (Takeda et al., 2001).

RUNX2 and histone deacetylase 4 (HDAC4) are expressed in prehypertrophic and hypertrophic chondrocytes present in developing cartilages. HDAC4 regulates chondrocyte differentiation and endochondral bone formation by interacting with and inhibiting the activity of RUNX2 (Inada et al., 1999; Enomoto et al., 2000; Vega et al., 2004). Furthermore, *Hdac4*-deletion mice exhibit precocious endochondral ossification of cranial synchondrosis (Vega et al., 2004). Myocyte enhancer factor 2C (MEF2C) regulates a *Runx2* enhancer in chondrocytes, and an activating form of MEF2C in mice causes precocious chondrocyte hypertrophy as well as ossification in SOS (Arnold et al., 2007). *Runx2* expression has been detected in the cartilaginous condensation of the cranial cartilages at embryonic day 13.5 (Funato et al., 2020), yet ossification of synchondroses did not occur in the wild-type embryos. This time lag between *Runx2* expression and execution of chondrocyte differentiation in the synchondroses implies that multiple layers of regulation are required in synchondrosis development and that HDAC4 and MEF2C could be the regulators involved in this process.

### T-Box Transcription Factor Family 1

T-box Transcription Factor Family 1 (*TBX1*) is the candidate gene of DiGeorge (OMIM #188400), velocardiofacial (OMIM #192430), and conotruncal anomaly face (OMIM #217095) syndromes. *Tbx1*-deficient mice exhibit most features similar to the human syndromes, including microcephaly (Jerome and Papaioannou, 2001; Lindsay et al., 2001; Funato et al., 2012, 2015). Mice lacking *Chrd—*which encodes chordin, i.e., an antagonist of bone morphogenetic proteins (BMPs)—exhibit recapitulating phenotypes of DiGeorge syndrome and *Tbx1*-deletion mice (Bachiller et al., 2003). Recently, we reported that TBX1 is a specific and essential regulator of chondrocyte differentiation and subsequent ossification at the SOS (Funato et al., 2020). By inhibiting the activity of RUNX2 and the expression of RUNX2 target genes, TBX1 negatively regulates chondrocyte differentiation and subsequent ossification in the SOS (Figure 1E).

### FGF Pathway

The FGF receptor (FGFR) family is a subfamily of receptor tyrosinekinases. Dominant gain-of-function mutations of *FGFR2* induce craniofacial dysmorphology in Apert (OMIM #101200), Crouzon (OMIM #123500), Pfeiffer (OMIM #101600), Jackson-Weiss (OMIM #123150), and Antley-Bixler (OMIM #207410) syndromes. Mice carrying the *Fgfr2* mutation exhibit accelerated chondrocyte maturation, accompanied by precocious ossification in the SOS and ISS synchondroses, at birth and 4 week-old stage, respectively (Nagata et al., 2011). A patient with Antley-Bixler syndrome was also identified to be harboring a mutation in *FGFRL1* (Rieckmann et al., 2009). *Fgfrl1-*deficient mice showed precocious ossification in the SOS at E18.5 (Catela et al., 2009). Homozygous mutations in the *POR* gene, encoding cytochrome P450 oxidoreductase, induce midface hypoplasia and craniosynostosis in Antley-Bixler syndrome, accompanied by genital anomalies and disordered steroidogenesis (OMIM #201750). Conditional deletion of *Por* in osteoprogenitors with *Dermo1-Cre* affects synchondrosis and long bone development in mice recapitulating Antley-Bixler syndrome (Panda et al., 2013). Although the craniofacial dysmorphology caused by *POR* mutations and by *FGFR2* mutations overlap, the pathogenesis underlying the skeletal malformation in *POR* deficiency remains to be elucidated.

Gain-of-function mutation of *FGFR3* is reported in most cases of achondroplasia (OMIM #100800) and Muenke syndrome (OMIM #602849), which are associated with craniofacial and skeletal abnormalities. Targeted mutations in *Fgfr3* in mice carrying the equivalent human syndromes lead to decreased chondrocyte proliferation along with accelerated osteoblast differentiation, resulting in precocious ossification of synchondroses (Chen et al., 1999, 2001; Matsushita et al., 2009; Laurita et al., 2011). Additionally, chondrocyte-specific expression of constitutively active mitogen-activated protein kinase 1 (MAP2K1)/MEK1 causes precocious ossification of cranial synchondroses and effectively rescues the *Fgfr3*-deficient mouse phenotype (Matsushita et al., 2009).

### PTHLH/PTHrP Pathway

PTHLH, also known as PTHrP, maintains chondrocyte proliferation in conjunction with parathyroid hormone 1 receptor (PTH1R). PTHLH/PTHrP impedes chondrocyte differentiation through the inhibition of *Runx2* expression (Li et al., 2004). In *Pthlh/Pthrp*-deletion mice, chondrocyte differentiation is accelerated in both the SOS as well as the ISS (Ishii-Suzuki et al., 1999). Moreover, PTHLH/PTHrP promotes dephosphorylation and nuclear localization of HDAC4, subsequently inhibiting MEF2C transcription (Kozhemyakina et al., 2009). *Pth1r*-deletion mice are shown to exhibit abnormal neurocranium morphology due to excessive mineralization of synchondroses present between the basioccipital, exoccipital, and basisphenoid bones (Lanske et al., 1996).

### Ihh Pathway

The Ihh pathway coordinates diverse aspects of bone morphogenesis via PTHLH/PTHrP-dependent and independent processes (St-Jacques et al., 1999). Ihh is expressed in the synchondroses within the prehypertrophic chondrocytes via RUNX2 regulation and promotes chondrocyte proliferation as well as differentiation (Young et al., 2006; Nagayama et al., 2008; Ushijima et al., 2014). In *Ihh*-deficient synchondroses, chondrocyte proliferation is decreased, and their differentiation is initially delayed (Razzaque et al., 2005; Young et al., 2006). Furthermore, conditional deletion of *Ihh* with *Col2a1-Cre* results in loss of the SOS at E18.5 (Razzaque et al., 2005).

### Cilium Assembly

The hedgehog signaling pathway requires cilium assembly. Kinesin family member 3A (KIF3A) is an intraflagellar transport (IFT) motor protein essential for the formation of cilia (Huangfu et al., 2003). Conditional deletion of *Kif3a* with *Col2a1-Cre* results in precocious ossification of synchondroses, by disrupting the expression pattern of *Ihh* in synchondroses (Koyama et al., 2007). Conditional deletion of *Ift88*, which encodes IFT88/polaris, ultimately results in a deformed basicranium, along with precocious ossification of synchondroses due to disruption of the Ihh signaling pathway (Ochiai et al., 2009). Polycystin-1 and polycystin-2, which are encoded by *Pkd1* and *Pkd2*, form a protein complex and localize to the primary cilium. Conditional deletion of *Pkd1* with *Dermo1-Cre* exhibits a premature closure of both the ISS and SOS (Kolpakova-Hart et al., 2008), whereas conditional deletion of *Pkd2* in neural crest with *Wnt1-Cre* exhibits abnormal ossification of neural crest-derived ISS (Khonsari et al., 2013).

*EVC* and *EVC2* are the disease genes implicated in Ellis-van Creveld syndrome (OMIM #225500) as well as Weyers acrofacial dysostosis (OMIM #193530). EvC ciliary complex subunit 1 (EVC) and EVC2 localize at the base of chondrocyte cilia and function as positive regulators of Ihh-mediated bone development (Takeda et al., 2002; Ruiz-Perez et al., 2007; Caparrós-Martín et al., 2013). Both *Evc*- and *Evc2*-deficient mice exhibit precocious ossification of the ISS at E18.5 (Pacheco et al., 2012; Caparrós-Martín et al., 2013).

ADP-ribosylation factor-like 6, which is encoded by *ARL6/BBS3*, regulates intracellular traffic. Mutations in *ARL6/BBS3* account for Bardet-Biedl syndrome-3 (OMIM #600151), which is characterized by retinal dystrophy, renal structural abnormalities, history of obesity, and skeletal abnormalities. *Arl6/Bbs3*-deficient mice are shown to exhibit hypomorphic cranial synchondroses at E18.5 (Kawasaki et al., 2017).

### Wnt/β-Catenin Pathway

The Wnt/β-catenin and Ihh signaling pathways interact with one another to regulate the development of the endochondral bones (Mak et al., 2006). Conditional deletion of *Ctnnb1*, which encodes CTNNB1/β-catenin, with *Col2a1-Cre* results in abnormal bone formation (Day et al., 2005; Nagayama et al., 2008). β-catenin and T-cell factor/lymphoid enhancer factor 1 (TCF/LEF1) are transcriptional mediators of the Wnt/β-catenin signaling pathway that directly interact with the *Ihh* promoter in chondrocytes *in vivo*, suggesting that the Wnt/β-catenin signaling pathway regulates *Ihh* expression (Später et al., 2006). Cartilage overexpression of a constitutively active form of LEF1 causes accelerated chondrocyte hypertrophy, topographical disorganization, and excessive bone collar formation in the ISS and SOS (Nagayama et al., 2008). Interestingly, LEF1 is reported to interact with and consequently inhibit the activity of RUNX2 (Kahler and Westendorf, 2003), suggesting that LEF1 might regulate RUNX2 activity during the development of synchondroses.

### SIX Homeobox Family

The sine oculis homeobox (SIX) family of transcription factors regulates the embryonic development of the ears and kidneys. *Six2*-deficient mice display precocious ossification of synchondroses at birth due to disruptions in chondrocyte differentiation, in conjunction with reduced proliferation and accelerated terminal differentiation of the cells (He et al., 2010). *SIX1* is implicated in Branchiootic syndrome 3 (OMIM #608389) and deafness (OMIM #605192). Double knockout mice of *Six1* and *Six4* genes show a precocious partial ossification of the SOS at E18.5 (He et al., 2010).

## Discussion

During synchondrosis development, the cross-talk between several signaling pathways, including PTHLH/PTHrP, FGF, Ihh, and Wnt/β-catenin, and control by cilium assembly and by transcription factors, play critical roles. Since the cranial abnormalities in female carriers of the P250R mutation in FGFR3 are more severe than those of the male carriers (Lajeunie et al., 1999), it would be interesting to study whether the onset and complete ossification of synchondroses vary based on gender in wild-type and genetically modified mice. Histological analysis of precocious ossification of synchondroses indicated that the deletion of the RUNX2 inhibitors HDAC4, MEF2C, and TBX1 in mice resulted in accelerated chondrocyte differentiation and, consequently, precocious endochondral ossification of cranial synchondroses (Vega et al., 2004; Arnold et al., 2007; Funato et al., 2020). Consistent with the precocious ossification of the synchondroses in these genetically modified mice, chondrogenic markers were ectopically expressed during synchondrosis formation. Since bone collar ossification occurs secondary to chondrocyte hypertrophy during endochondral bone formation (Chung et al., 2001; Arnold et al., 2007), precocious ossification of synchondroses in these genetically modified mice could occur when chondrocyte hypertrophy is accelerated. The accelerated chondrocyte hypertrophy may also result in a shortage of the reserves of resting and proliferating chondrocytes.

### Phenotypic Differences Between SOS and ISS

The synchondrosis phenotype is different among genetically modified mice. Deletion of *Pthlh/Pthrp* or *Six2* or overexpression of *Runx2* in chondrocytes resulted in precocious ossification both in the ISS and the SOS. Precocious ossification is specific to the SOS in *Tbx1*-, *Fgfrl1*-, *Ihh*-, and *Pth1r-*deficient mice and *Mef2c*-superactivating mice, whereas it is specific to the ISS in *Arl6/Bbs3-*, *Evc*-, and *Evc2-*deficient mice (Table 1). Phenotypic differences among the synchondroses may be due to varying origins of the ISS and SOS (Figure 1C). The cartilage primordium of the ISS is derived from the neural crest, whereas the SOS has a more complex origin, comprising the cartilage primordium derived from the neural crest along with the cranial mesoderm (McBratney-Owen et al., 2008). TBX1 is a specific regulator of SOS development. Since TBX1 is expressed in the mesoderm-derived primordium cartilage of the SOS, differences in the expression pattern of TBX1 likely contribute to the discordant abnormalities between the ISS and SOS (Funato et al., 2020). A consequence of functional redundancy of family genes might also contribute to the same. In the synchondroses of *Ihh*-deficient mice, the hypertrophic chondrocytes in the ISS are more affected than those in the SOS (Young et al., 2006). The remnants of the notochord express Sonic hedgehog (Shh) near the primordium of the SOS but not in the ISS. Since Shh has a redundant interaction with Ihh (Zhang et al., 2001), Shh may induce the milder phenotype of the SOS than the ISS of *Ihh*-deficient mice (Young et al., 2006).

### Phenotypic Differences Between the Growth Plate and Synchondroses

The growth plates of cranial synchondroses and long bones contribute to bone elongation as well as shaping of the mature bone via endochondral ossification. However, the growth plate of synchondrosis and the long bone are histologically, environmentally, and developmentally different in the following aspects: (1) the mirror image growth plates of synchondrosis produce longitudinal bone growth in bipolar directions, but the growth plate of long bones produces growth in unipolar direction; (2) the long bones are overlaid by articular synovial layers, which are absent in the synchondrosis; (3) the growth plate in developing long bones present the secondary ossification center, which is absent in the synchondrosis; (4) mechanical stress influences the growth of long bones (Sharir et al., 2011); and (5) the ISS originates from the neural crest, while the SOS has a complex unique contribution of both the neural crest and cranial mesoderm, and long bones are derived from mesoderm. Therefore, discordant abnormalities in the growth plates of the long bones and synchondroses are likely attributable to the differences in location-specific downstream signaling targets and the expression patterns of the signaling factors, which differ according to the unique origins and anatomical structures.

RUNX2, HDAC4, and MEF2C control endochondral ossification in the growth plates of both synchondroses and long bones (Takeda et al., 2001; Vega et al., 2004; Arnold et al., 2007). However, in other mutant mice, discordant abnormalities between long bones and synchondroses have been reported. Zinc finger transcriptional coregulator 521 (ZFP521), whose expression is regulated by PTHLH/PTHrP, associates with and antagonizes RUNX2 activity in chondrocytes via an HDAC4-dependent mechanism (Correa et al., 2010). Deletion of *Zfp521* in chondrocytes does not affect the synchondrosis development; however, long bones appear to be hypomorphic (Correa et al., 2010). Deletion of *Tbx1* results in precocious endochondral ossification of the SOS, but not in the skeletal cartilages despite TBX1 expression in immature chondrocytes (Funato et al., 2015, 2020).

In the synchondroses of *Pthlh/Pthrp-*deletion mice, chondrocyte differentiation is significantly accelerated compared with those chondrocytes present in long bones (Ishii-Suzuki et al., 1999). Ihh is expressed in prehypertrophic chondrocytes and stimulates *Pthlh/Pthrp* expression in periarticular chondrocytes in long bones. In the synchondrosis, an overlaid periarticular layer is absent, and the Ihh signaling relays cross-talks between Ihh-producing prehypertrophic chondrocytes and PTHLH/PTHrP-producing proliferating chondrocytes (Young et al., 2006). Since PTHLH/PTHrP is expressed in both the resting and the proliferating chondrocytes in the synchondroses and in the resting chondrocytes of long bones, varied distribution of PTHLH/PTHrP-expressing chondrocytes may contribute to the discordant phenotypes between the synchondrosis and long bones of *Pthlh/Pthrp-*deficient mice (Young et al., 2006; Nagayama et al., 2008).

## Conclusion

Synchondroses are formed through endochondral ossification and play a critical role in the elongation of the basicranium. Deletions or activation of genes can cause the precocious ossification or hypoplasia of synchondroses, suggesting that stringent regulation of signaling pathways is crucial for proper synchondrosis development. The disruption of genes leads to both similar and distinctly different abnormalities in the development of the two synchondroses and also between the growth plates of synchondrosis and skeletal bones. Despite its importance, few studies have addressed the molecular mechanisms that regulate the endochondral ossification of synchondroses. It is important to fully elucidate the interaction of signaling pathways for the regulation of synchondrosis development. In addition, the detailed molecular mechanisms that mark the differences between the synchondroses and the skeletal bones should be deciphered. Hopefully, these insights from future studies will provide possible strategies for biologics-based therapies to treat synchondrosis anomalies.

## Author Contributions

NF contributed to the conceptual idea, performed the database searches, analyzed the data, and wrote the manuscript.

## Conflict of Interest

The author declares that the research was conducted in the absence of any commercial or financial relationships that could be construed as a potential conflict of interest.

## Footnotes

1 http://www.informatics.jax.org

2 http://www.ncbi.nlm.nih.gov/pubmed

3 http://string-db.org
